# Detection of Viruses with Oncogenic and Oncomodulatory Potential in Head and Neck Tumors—External Auricle

**DOI:** 10.3390/biomedicines13102339

**Published:** 2025-09-25

**Authors:** Kalina Shishkova, Ivo Sirakov, Stoyan Shishkov, Elena Tasheva-Terzieva, Stefan Dimitrov Gergov, Zornitsa Tileva, Reneta Dimitrova, Ivailo Alexiev, Raina Gergova

**Affiliations:** 1 Laboratory of Virology, Faculty of Biology, University of Sofia “St. Kl. Ohridski”, 8 Dragan Tzankov Blvd., 1164 Sofia, Bulgaria; sashishkov@yahoo.com (S.S.); ztileva@gmail.com (Z.T.); 2 Department of Medical Microbiology, Faculty of Medicine, Medical University of Sofia, 2 “Zdrave” Str., 1431 Sofia, Bulgaria; insirakov@gmail.com (I.S.); rtgergova@gmail.com (R.G.); 3 Department of Zoology and Anthropology, Faculty of Biology, University of Sofia “St. Kl. Ohridski”, 8 Dragan Tzankov Blvd., 1164 Sofia, Bulgaria; elena.tasheva@gmail.com; 4 Department Head and Neck Surgery, National Oncological Hospital “Ivan Chernozemski”, 1504 Sofia, Bulgaria; s_gergov@mail.bg; 5 National Reference Laboratory of HIV, National Center of Infectious and Parasitic Diseases, 1504 Sofia, Bulgaria; naydenova.reneta@gmail.com (R.D.); ivoalexiev@yahoo.com (I.A.)

**Keywords:** co-infection, external auricle, cancer, oncogenic viruses

## Abstract

It is estimated that approximately 90% of all head and neck cancers are squamous cell carcinomas with a complex and multifactorial etiology. Molecular and epidemiological studies provide evidence for the role of oncogenic viruses in the initiation and/or oncomodulation of head and neck squamous cell carcinoma. **Objectives**: The present study aimed to detect the presence of high- and low-risk HPV, BKPyV, EBV, HCMV, HSVs in biopsy samples of squamous cell carcinoma of the external auricle in patients in Bulgaria. **Materials and Methods:** The study included 41 biopsy specimens from etiologically undiagnosed cases of squamous cell carcinoma of the external auricle. Molecular biological methods were used to detect the viruses—conventional and nested PCR, and sequence analysis. **Results:** The results obtained showed that none of the samples were found to have a high-risk HPV genotype. The highest percentage of samples showed genotype 6/11, and the lowest number of samples showed low-risk genotype 44. Of all herpesviruses, EBV was found in the largest proportion of samples, being present in the sample as a co-infection with HPV and always together with genotype 6/11. The frequency distribution, as a percentage and number of samples, of the possibilities for co-infection of EBV with each of the HPV genotypes was established. Of the remaining herpesviruses, the presence of HSV 2 was not confirmed in any of the samples. HSV 1 was present in only three of the samples, as a co-infection with genotypes 6/11, 42 and 43. When examining the samples for the presence of HCMV, only one positive sample was found, with both HPV 6/11 and 42 additionally present in the sample. **Conclusions:** For the first time, HPV, BKPyV, EBV, HCMV and HSVs were investigated and their possible involvement alone or as co-infection in the carcinogenesis of squamous cell carcinoma of the external auricle in patients in Bulgaria. The presence of the mentioned viruses, as well as the non-random distribution of EBV + HPV 6/11 and EBV + HPV 44, proven by us, does not necessarily make them etiological agents, but they could, through different and known mechanisms, influence the initiation and/or modulation of carcinogenesis.

## 1. Introduction

Head and neck cancer (HNC) is responsible for over 500,000 cases, including over 300,000 deaths, annually worldwide [1]. It has been estimated that approximately 90% of all head and neck cancers are squamous cell carcinoma (SCC). The etiology of SCC is complex and involves a number of factors. Molecular, epidemiological, and virological studies provide evidence for the role of oncogenic viruses in the development of SCC [2].

The human papillomavirus is a well-studied etiological agent associated with oncological diseases, and a number of authors have reported the relationship between these viruses and the development of head and neck cancer [3]. Basal epithelial cells are susceptible to HPV infection, although only upper highly differentiated cells in the stratified epithelium are permissive [4,5]. A key part in the potential role of HPV in the malignant transformation of cells is played by the oncogenes E6 and E7, which inactivate the tumor suppressors p53 and Rb—thus causing uncontrolled cell growth [6,7]. Human polyoma BK virus (BKPyV), a member of the Polyomaviridae family, has been associated with human tumors and is classified as a potential human carcinogen [8,9]. It is estimated that 90% of the population is infected with BK virus, probably in early childhood. Viral DNA has been detected in tumors such as neuroblastoma, urinary tract tumors, carcinomas of the cervix and vulva, lip and tongue, and Kaposi’s sarcoma [10,11,12]. According to the authors, BKPyV is among the main causes of prostate and bladder cancer. Additional localization of viral DNA has also been demonstrated in the salivary glands. The large T-antigen expressed by polyomavirus, similar to HPV oncoproteins, can interact with p53 and pRB, thereby overcoming cell death mechanisms [13,14]. Herpes simplex viruses types 1 and 2 are less associated with carcinogenesis, but they can establish latent infections in nerve ganglia due to the so-called LAT transcripts. Although these simplex viruses are not considered oncogenic viruses in their own right, they can interact with other viruses associated with cancer development such as Epstein–Barr virus or human papillomavirus [15]. In addition, HSV-2 may play a role as a co-factor for HPV-associated neoplasia by inducing chronic inflammation [16]. Also, HSV may be involved in coinfection with HPV in some immunodeficiency diseases such as Hyperimmunoglobulin E syndrome [17].

EBV, like HPV, exhibits epithelial tropism, but EBV entry into cells occurs via both apical and basolateral routes into the mucosal epithelium [18]. This virus shows an additional and preferential tropism for B cells, which constitute the EBV reservoir [18]. The virus, like other herpesviruses, establishes latent infection in memory B cells and contributes to oncogenesis primarily through the expression of latent genes that interfere with normal cellular processes, such as cell cycle regulation, apoptosis, and immune surveillance. LMP1 is the major oncoprotein of EBV, functioning as a mimic protein of the tumor necrosis factor receptor family member CD40. Epstein–Barr virus DNA has been detected in samples of temporal bone squamous cell carcinomas, with the authors suggesting a possible etiological link [19], but mainly the virus is associated with development of nasopharyngeal and salivary gland cancers. Several cases of EBV-positive middle ear carcinoma have been diagnosed, which is an extremely rare tumor, histologically resembling nasopharyngeal carcinoma, and can be assumed to be part of the group of EBV-associated tumors [20].

HCMV is a pathogen that causes mainly opportunistic infections associated with severe illness and mortality in immunocompromised individuals, transplant recipients, cancer patients, and AIDS patients. Approximately 70–90% of the world’s population is infected with the virus, which, like other members of the herpesvirus family, remains latent throughout the host’s life after primary infection. Cytomegalovirus, like EBV, is not considered a direct oncogenic virus. It is assumed that it acts as an oncomodulator, i.e., it promotes tumor development and progression without directly transforming cells. The oncomodulatory effect is due to the virus’s mechanisms for escaping the immune response, such as mimicking IL-10 and 6, maintaining chronic inflammatory processes in the area around the tumor by modulating the immune response, blocking apoptosis, etc [21]. HCMV infection in humans causes the progression and development of several types of cancer, including female breast cancer [22], brain cancer [23] and colorectal cancer [24]. Several studies have revealed that the HCMV genome contains oncogenes—*US28*, *IE1*, *IE2*, *UL76* and *UL 82* [25,26,27] suggesting an important role for HCMV in cancer progression. HCMV infection has been shown to activate key signaling pathways associated with cancer [28].

The present study aims to detect the presence of high- and low-risk HPVs, BKPyV, EBV, HCMV, HSVs in samples of squamous cell carcinoma of the external auricle in patients in Bulgaria. Since data on the possible involvement or presence of these viruses in squamous cell carcinoma of the external auricle are relatively scarce, we set ourselves the task of establishing both their presence in this type of tumor and the likelihood that they are related to the disease alone or in the form of co-infection.

## 2. Materials and Methods

### 2.1. Samples

The study included 41 biopsy specimens of etiologically undiagnosed squamous cell carcinoma cases of the external ear. All tumors were on the auricle, mainly the upper outer part. Pathohistological examinations of all samples showed Squamous cell carcinoma—which is rare and moderately malignant—between the squamous cell and basal cell. The patients were predominately (38) men over the age of 60 and only three women. The history revealed that the patients had been exposed to external factors for a long time—sun, wind, etc. The samples were obtained in the period 2014–2015, according to the diagnostic process, and stored at −80 °C.

### 2.2. DNA Extraction Procedures

For DNA isolation we used a Quick-DNATM Miniprep Plus extraction kit (Zymo Research Corporation, Tustin, CA, USA). Samples were processed according to the manufacturer’s instructions.

### 2.3. Molecular Methods for Detection of the Viruses Included in the Study

For detection of human beta globin gene, HSV1 and 2, BKPyV, HCMV, EBV and HPV low risk genotypes 6/11, 42, 43, 44 we used conventional PCR and primers described in Table 1. The HPV detection reactions were performed in two stages: first PCR with GPE primers, and then nested PCR with the primers for the individual genotypes.

To perform the PCR reactions, we used Ruby Hot Start Master (2×) (Jena Bioscience, Jena, Germany). The standard PCR and the first nested PCR reactions were performed as follows: 10 μL PCR Master mix, 6 μL PCR water (Jena Bioscience, Germany), 2 μL extracted DNA and 2 μL of primer’s mix which was made from 10 pmol of each primer. When performing the nested PCR, we used 0.5 μL of the first PCR reaction, 10 μL PCR Master mix, 2 μL of primer’s mix which was made from 10 pmol of each primer and 7.5 μL PCR water (Jena Bioscience, Germany). In the negative controls, instead of DNA, the same amount of PCR water (Jena Bioscience, Germany) was added to the reaction mixture. The quality of the DNA extraction and PCR products were controlled by gel electrophoresis in 2% agarose gel (Bioline, Meridian Bioscience, Memphis, TN, USA), 1× TAE buffer, a 10 pmol/mL concentration of Ethidium bromide (Sigma-Aldrich, Steinheim, Germany), and 100 bp and 1 kb DNA markers (Bioline, Meridian Bioscience, Memphis, TN, USA).

For detection of 19 high-risk HPV (16, 18, 26, 31, 33, 35, 39, 45, 51, 52, 53, 56, 58, 59, 66, 68, 69, 73 and 81) we used a combination of PCR and hybridization of E6 and E7 regions of the virus, using High PapillomaStrip 16 kits (OPERON, Zaragoza, Spain). The protocol was applied according to the manufacturer’s instructions. For detection of HCMV, a kit from Amplisens (Ecoli Dx, s.r.o., Praha, Czech Republic) was used.

The annealing temperatures of the primers were checked and corrected using the QB96 apparatus program. All sequences of the primers used were checked in Blastn NCBI (National Library of Medicine, Bethesda, MD, USA).

To eliminate the possibility of cross-contamination associated with nested PCR, they were performed in different Class II Biological Safety Cabinets with different sets of automatic pipettes, sterile filter tips and on different days.

#### Sequencing

Amplicons generated by PCR were purified using an enzymatic method with the PCR Clean-up reagent (Thermo Fisher Scientific, Cat. No. K310001, Waltham, MA, USA), which contains two hydrolytic enzymes: Exonuclease I and Alkaline Phosphatase. The reaction was performed under the following thermal conditions: 37 °C for 15 min, followed by enzyme inactivation at 80 °C for 15 min, then held at 4 °C indefinitely.

Cycle sequencing reactions were carried out using the BigDye™ Terminator v3.1 Cycle Sequencing Kit (Applied Biosystems, Thermo Fisher Scientific, Cat. No. 4337455) with the following thermal profile: initial denaturation at 96 °C for 1 min; 25 cycles of 96 °C for 10 s, 50 °C for 5 s, and 60 °C for 4 min; final hold at 4 °C.

Sequencing reaction products were purified using a sodium acetate/ethanol/EDTA precipitation method, following the manufacturer’s protocol (Applied Biosystems, 2002, Waltham, MA, USA). Purified products were resuspended in 20 μL Hi-Di™ Formamide (Thermo Fisher Scientific, Cat. No. 4311320), denatured at 95 °C for 2 min, and placed on ice. Capillary electrophoresis was performed using the Applied Biosystems 3500xL Genetic Analyzer (Thermo Fisher Scientific).

The resulting sequence data were initially analyzed using standard analysis software and databases. Additionally, Geneious Prime software (version 2021.1.1; Biomatters Ltd., Auckland, New Zealand) was used to further verify the generated sequences, assess read quality, and confirm base calls.

### 2.4. Statistical Analysis

The detection of each virus was calculated both as a proportion of infected patients and as a percentage of the total number of individuals tested. A 95% confidence interval (CI) was calculated for each proportion, using the Wilson score method, which provides an accurate estimate of the interval.

To assess the distribution of coinfections between Epstein–Barr virus (EBV) and each of the four low-risk HPV types (6/11, 42, 43, and 44), the McNemar test was used. This test is appropriate for paired binary data and assesses whether the proportions of discordant pairs differ significantly within the same patient group. Since multiple comparisons were performed, *p*-values were adjusted using the Bonferroni correction to control the family-wise error rate. A significance level of 0.05 was adopted for statistical tests.

## 3. Results

### 3.1. HPV

Of the 41 patients studied, 38 (92.7%) were male and 3 were female, or 7.3%. When the samples were tested, none of them were found to be positive for high-risk HPV genotypes. Of all the patients studied, 36 (87.8%) had one or more low-risk HPV genotypes, and only 5 (12.2%) patients were negative for papillomaviruses. Since the presence of more than one genotype was reported in some of the samples, the frequencies in the number of patients and the percentage of samples with positive tests for one or more HPV genotypes were calculated. The results are shown in Table 2 and Table 3.

From the results presented in Table 2, it is evident that the most common genotype is 6/11. Of all 41 samples, 33 or 80.5% are positive for it, and the smallest number of samples tested positive for genotype 44.

Given the fact that more than one genotype was found more often in the samples, the number and percentage of them in which they were registered were calculated. The results presented in Table 3 show that in five of the patients all four genotypes were found. The most common combination of genotypes in the study was 6/11, 42 and 43—they were found in 29.3% of the patients studied. Eleven patients were carriers of HPV genotype 6/11 only, and only one patient was found with genotype 42. It is noteworthy that in the majority of patients the combination of 6/11 and 42 was present.

### 3.2. Viruses from the Herpesvirus Family

Of the herpes viruses studied, EBV was found in the largest number of samples—17 or 41.5% of the samples were positive. Since more than one herpes virus was found in some of the probits, the total number of patients carrying any of the herpesviruses studied was 18 or 43.9% of the total number of those studied (Figure S1). Another reported fact was that all samples positive for any of the herpesviruses also contained HPV 6/11. The frequency distribution of the number of patients infected with more than one herpes virus was also analyzed. The results are presented in Table 4 and Table 5.

None of the samples showed the presence of HSV 2, only HSV 1 in three of the samples. Only one patient tested positive for HCMV.

Of the patients with coinfection with two herpes viruses, two had HSV 1 and EBV, and one had HCMV and EBV.

### 3.3. BKPyV

When examining the samples, by applying a conventional PCR method for detection of BKPyV, the size of the expected fragment was 1289 bp. After performing nested PCR to detect BK Polyomavirus, we obtained very distinct and strong fragments with a smaller size (~590 bp) than expected (Figure S2). To determine the origin/source of these fragments, we performed sequencing of these PCR products. After sequencing, we found no signal or it appeared as background noise. The majority of PCR-nonspecific samples did not generate readable sequence data, a common challenge observed when target template concentrations are low or when amplicons exhibit significant secondary structure that impedes polymerase progression during sequencing reactions. Furthermore, the few samples that did yield signals exhibited highly ambiguous chromatograms, characterized by superimposed and unresolved peaks (Figure S3). This is the reason why we did not include in the statistical processing of the results obtained for the BK virus.

In the statistical processing of the results, in addition to frequencies such as number of samples and %, 95% confidence intervals were also calculated. The summarized results are presented in Table 6.

In the entire study group of patients with squamous cell carcinoma of the external auricle (*n* = 41), infection with low-risk human papillomavirus (HPV LR) affected 87.8%. The most frequently detected genotype was HPV 6/11, detected in 80.5% of patients (95% CI: 66.0–89.8%), followed by HPV 42 (61.0%), HPV 43 (46.3%), and HPV 44 (12.2%).

**Figure 1:**
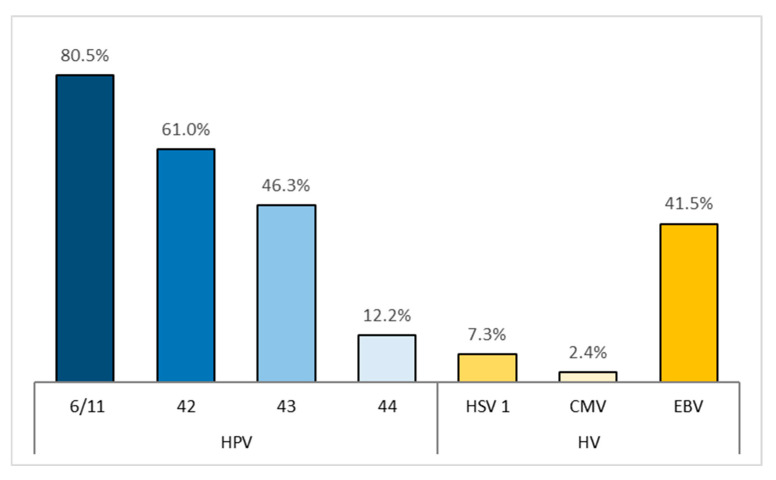
Prevalence of infections with low-risk human papillomavirus types (HPV 6/11, 42, 43, and 44) and herpesviruses (HSV-1, CMV, and EBV).

Herpesvirus (HV) infections were detected in 43.9% of patients. Among them, Epstein–Barr virus (EBV) showed the highest prevalence (41.5%, with 95% CI: 27.8–56.6%), while HSV-1 and HCMV were detected in 7.3% and 2.4% of patients, respectively, and a confidence interval was not calculated. The results are graphically illustrated in Figure 1.

The presented graph shows a significantly higher prevalence of HPV genotypes compared to the presence of herpesviruses in the samples.

**Figure 2:**
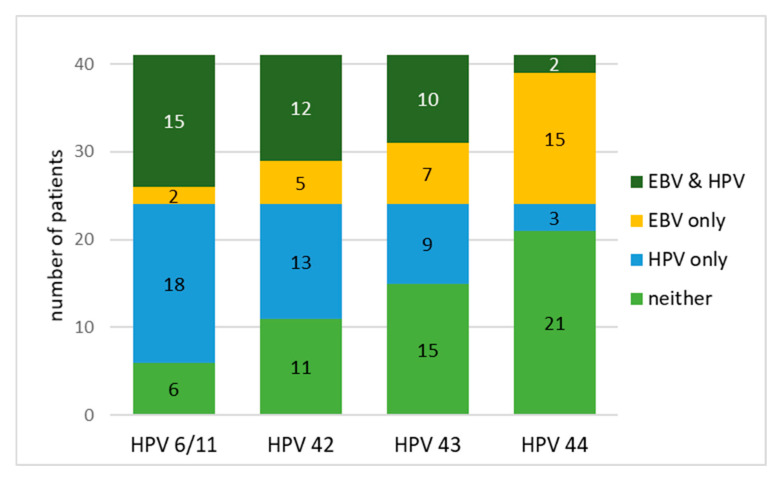
Distribution of EBV and HPV infections among patients.

McNemar’s test was used only for the HPV LR and EBV pair, since only for this pair are there enough positive samples to determine whether or not there is a risk of coinfection. The distribution of Epstein–Barr virus (EBV) and low-risk human papillomavirus (HPV) infections among patients is presented in Table 7 and Figure 2. The test was applied to assess whether the detection of EBV was significantly discordant with the detection of specific HPV types in the same patients.

Since multiple comparisons were performed (EBV against four HPV types), the resulting *p*-values were adjusted using the Bonferroni correction to control for multiple testing.

From the presented results, we can summarize that a significantly higher number of patients were positive for HPV 6/11 only compared to those positive for EBV only (43.9% vs. 4.9%), while 36.6% of patients were positive for both viruses. McNemar’s test only reports % mismatches (EBV+/HPV- and EBV-/HPV+). The test revealed a statistically significant difference between the paired results and the separate results (χ^2^ = 11.3, *p* < 0.004), indicating a non-random pattern of co-detection.

For HPV 42, although co-presence was observed in 29.3% of patients and HPV 42 infection alone in 31.7%, the discrepancy did not reach statistical significance (χ^2^ = 2.72, *p* = 0.396). Similarly, HPV 43 did not show a significant discrepancy in detection rates (χ^2^ = 0.06, *p* = 1.000), with all four categories being comparably distributed.

In contrast, HPV 44 showed a significant imbalance, with EBV-only infections observed in 36.6% of patients, while only 7.3% were positive for HPV 44 alone and 4.9% for both viruses. McNemar’s test confirmed a significant disparity (χ^2^ = 15.4, *p* = 0.038), suggesting a lower probability of HPV 44 and EBV coinfection.

The results showed virus type-specific patterns in the co-occurrence of EBV and low-risk HPV types, with significant asymmetry observed for HPV 6/11 and HPV 44.

## 4. Discussion

Squamous cell carcinoma (SCC) of the external auricle is a malignant tumor arising from the squamous cells of the skin of the external ear. It is the second most common type of skin cancer after basal cell carcinoma, but SCC of the ear is more aggressive, with a higher risk of recurrence and metastasis than in other areas. Sun exposure, radiation exposure, and chronic infection, as well as the association with HPV-induced viral carcinogenesis, are among the predisposing factors.

Pathogen-associated malignancies may result from the establishment of prolonged latency as a consequence of chronic infections [1]. Pathogenic infections alone are not a sufficient factor for the initiation or progression of tumor disease [1,2,3]. A number of studies available in the literature address the role of viruses in the development of head and neck squamous cell carcinoma (HNSCC). Most of them have focused mainly on one type of virus. Our study aims to detect viruses with possible oncogenic or oncomodulatory potential in samples of squamous cell carcinoma of the external auricle in patients from Bulgaria, while also analyzing the possible correlation between infection with more than one oncogenic virus and carcinogenesis or modulation of the oncogenic process.

We investigated the presence of HPV high- and low-risk genotypes, BKPyV, EBV, HCMV and human herpes simplex viruses (HSVs). The probability distribution of frequencies and the possibilities of co-infection between them were also analyzed.

It was found that of all the HPV genotypes studied—listed in the Section 2—only low-risk ones were present in the samples. At least one genotype was detected in 87% of the samples. Strikingly, genotype 6/11 was the most common—it was present in the largest percentage of positive samples for the virus. The most common combination of genotypes in the samples was 6/11, 42 and 43—they were detected in 29.3% of the patients studied.

The obtained result correlates to some extent with studies by teams of scientists who believe that there is no obligatory relationship between HPV infection with high-risk genotypes and neoplastic progression of squamous cell carcinoma [37,38,39,40,41,42]. The presence of low-risk HPV, especially genotypes 6 and 11, in various HPV-associated lesions has been confirmed by different teams who claim that HPV 6 and 11 in the absence of high-risk genotypes can sometimes be associated with high-grade lesions [43]. It is assumed that interactions between high- and low-risk HPV types reduce the risk of squamous cell carcinoma of the cervix, and the results for high-risk genotypes in our samples were negative and this is perhaps another reason to assume the association of low-risk HPV genotypes with squamous cell carcinoma [44].

The association between juvenile recurrent respiratory papillomatosis (RRP) and low-risk human papillomaviruses (HPV) types 6 and 11 has been demonstrated.

The team identified a new molecular mechanism underlying the first case of HPV6-associated laryngeal carcinoma in juvenile RRP, i.e., that HPV6 integration into the aldo-ketoreductase 1C3 gene leads to loss of its expression, and changes in the expression of this gene are associated with the tumorigenesis of other (HPV-related) malignancies. Our results confirm the results obtained by other groups, namely that through different mechanisms low-risk HPVs could have an impact on the processes of initiation and/or modulation of carcinogenesis [45].

In experiments to detect BKPyV, to reduce the possibility of amplifying nonspecific PCR products, we used Hot Start PCR [46,47,48]. However, in the study of BKPyV, we obtained the results described in the Section 3. We used sequencing to identify the resulting fragments [49]. Despite the expectation that the product was the result of the amplification of a mutated genomic region of the neoplasia, the sequencing result was negative. The reasons for the formation of nonspecific PCR fragments are indicated as the formation of primer dimers [50,51], low concentration of the target gene [52], low temperature prior to the first denaturation step [46,47,48]. The PCR procedures applied and described by us in the Section 2 were performed in a manner (primer verification, negative controls, nested PCR and Hot Start PCR) which suggests a reduction in the possibility of the appearance of nonspecific products based on the reasons described above. In this regard, the amplification of non-specific PCR products in BKPyV may most likely be the result of the so-called phenomenon dubbed “jumping”, which is a result of the fact that in the presence of extended primers and if they share homology with a sequence elsewhere in the genome, an entirely new product is amplified during intragenomic recombination [53,54]. In our study of BKPyV, only the internal primer BKV-VP1-F4 is 25 nucleotides long, and the others are 20 nucleotides long, but considering that we are studying biopsy material from neoplastic tissue, it is entirely possible that the so-called “jumping” is the cause of the formation of non-specific fragments. It is assumed that the frequency of jumps depends on the ratio of template to non-template DNA, and not on the absolute concentration of the template [54].

In all three of the positive HSV 1 samples the virus was in combination with 6/11, 42 and 43, which confirms the assumptions of the author’s teams for a possible synergistic effect of the herpes virus with HPV. Martyn Cox et al. [55] suggest such a synergistic interaction of HSV1 and a high-risk genotype of papillomavirus, as a likely early event and HSV may be detected more often in potentially malignant lesions than in carcinoma.

As can be seen from the presented results, only EBV with HPV genotypes were analyzed for the presence or absence of coinfections, since only for these pairs of viruses were statistically reliable analyses available. The statistical analysis revealed a significant discrepancy in the detection of EBV and two of the low-risk HPV types, 6/11 and 44. For HPV 6/11, a higher number of patients were positive for HPV alone compared to the number of patients infected with EBV alone, with coinfection being relatively frequent (*p* < 0.001). A different pattern was observed for HPV genotype 44, where EBV-only infections were significantly more frequent than coinfections or HPV-44-only infections (*p* = 0.010). This opposite trend suggests a different and non-random distribution profile for HPV 44 compared to the other HPV types studied. For the other two genotypes, the distribution was random, as there were no significant differences between positive, negative samples and their combination with EBV.

No statistically significant discrepancy was found, which is indicative of the McNemar’s test, for HPV 42 and HPV 43 (*p* = 0.396 and *p* = 1.000, respectively), indicating more symmetrical detection patterns such as coinfection with EBV. Overall, the results indicate type-specific differences in co-detection profiles, with HPV 44 showing a contrasting trend compared to HPV 6/11, 42, and 43.

The potential mechanisms of HPV/EBV interaction remain unclear, although some possibilities have been proposed. HPV may facilitate EBV entry by stimulating increased expression of integrins and CD21, and promote the establishment of EBV latency by inducing genetic changes due to integration of the HPV genome into the host cell genome. Mechanisms of escape from the immune response stimulated by HPV infection may facilitate secondary EBV infection in epithelial cells. Furthermore, HPV and EBV viral oncoproteins may cooperate synergistically in head and neck carcinogenesis [56].

The limitation of the study is related to the small number of samples, which is relevant and related to the small size of the Bulgarian population and the fact that it is an oncological, rare disease (and not an infectious respiratory disease), homogeneity of the cohort (mostly men over 60 years of age, but these are all patients in the country who sought help and were diagnosed with such a disease), omission of some clinical and epidemiological variations (e.g., smoking status, immunosuppression, concomitant infections). Given the fact that the group is small, due to the reasons listed above, such a division of patients into subgroups would not allow any significant conclusions to be drawn. There are no negative controls because the procedure is invasive and not applied to healthy people.

## 5. Conclusions

For the first time, HPV, BKPyV, EBV, HCMV and HSVs were investigated and their possible involvement alone or as co-infection in the carcinogenesis of squamous cell carcinoma of the external auricle in patients in Bulgaria. The development of this type of tumor is a multifactorial process. The unlocking/unblocking of cellular oncogenes can also be due to physical and chemical factors. The presence of high-risk HPV genotypes was not proven. Low-risk genotypes were found in the majority of samples, with 6/11 prevailing.

When examining the probability of coinfection between EBV and each of the HPV genotypes, significant and reliable differences were observed in the detection of EBV and two of the low-risk HPV types, 6/11 and 44. For HPV 6/11, a higher number of patients were positive for HPV alone compared to the number of patients infected with EBV alone, with coinfection being relatively common (*p* < 0.004). A different pattern was observed for HPV genotype 44, where EBV-only infections were significantly more common than coinfections or HPV 44-only infections (*p* = 0.038). This inverse trend suggests a different and non-random distribution profile for HPV 44 compared to the other HPV types studied. For the other two genotypes, the distribution was random, as there were no significant differences between positive, negative samples, and their combination with EBV. The presence of the mentioned viruses, as well as the non-random distribution of EBV + HPV 6/11 and EBV + HPV 44, proven by us, does not necessarily make them etiological agents, but they could, through different and known mechanisms, influence the initiation and/or modulation of carcinogenesis.

## Figures and Tables

**Table 1:** Description of the primers used.

Primers				Annealing	Source
Target/Genes	Name	Sequence 3′-5′	Fragment Size bp	Temperature °C	
HSV1/US4(gpG)	F HSV1	CTGTGGTGTTTTTGGCATCA	123	62	[29]
	R HSV1	GGTTGTGGAGGAGACGTTG			
HSV2/US6 (gpD)	F HSV2	CATGGGGCGTTTGACCTC	249	62	
	R HSV2	TACACAGTGATCGGGATGCT			
BKPyV/BKPyVgp3	BKV-VP1-F1	AAACTATTGCCCCAGGAGGT	1289	57	[30,31]
	BKV-VP1-R4	CTAAAACACCACCCCCAAAA			
	BKV-VP1-F4	CTAATCAAAGAACTGCTCCTCAATG	1261	57	
	BKV-VP1-R8	ACCACCCCCAAAATAACACA			
EBV/EBNA-1	EBV1n	ATCGTGGTCAAGGAGGTTCC	209	53	[32]
	EBV2n	ACTCAATGGTGTAAGACGAC	209		
	EBV3	AAGGAGGGTGGTTTGGAAAG	297	54	
	EBV4	AGACAATGGACTCCCTTAGC	297		
HPV/E6 and E7	GP-E6-3F	GGGWGKKACTGAAATCGGT	630–700	55	[33]
	GP-E6-5B (R)	CTGAGCTGTCARNTAATTGCTCA			
	GP-E6-6B (R)	TCCTCTGAGTYGYCTAATTGCTC			
	6/11F	TGCAAGAATGCACTGACCAC	334	56	[34]
	6/11R	TGCATGTTGTCCAGCAGTGT			
	42F	CCCAAAGTAGTGGTCCCAGTTA	277		
	42R	GATCTTTCGTAGTGTCGCAGTG			
	43F	GCATAATGTCTGCACGTAGCTG	219		
	43R	CATGAAACTGTAGACAGGCCAAG			
	44F	TAAACAGTTATATGTAGTGTACCG	163		
	44R	TATCAGCACGTCCAGAATTGAC			
Human beta globin	GH20 **	GAAGAGCCAAGGACAGGTAC	268	52.9–60.3 [33]	[35,36] *
	PC04	CAACTTCATCCACGTTCACC			

* Primers for beta globin according to Saiki et al. [35] and Ritari et al. [36]; gpG—glycoprotein G; gpD—glycoprotein D; ** elongation time—for the external primers of HPV and BKPyV this is 60 s, for all others it is 30 s.

**Table 2:** Frequencies in number and % of patients with positive tests.

	Patients	
	Number	%
**HPV LR**	**36**	**87.8%**
6/11	33	80.5%
42	25	61.0%
43	19	46.3%
44	5	12.2%

**Table 3:** Frequency in number and % of samples with different numbers of positive tests for individual papillomavirus genotypes.

Number of Genotypes	Patients	
	Number of Samples	%
4	5	12.2%
3	12	29.3%
2	7	17.1%
1	12	29.3%
0	5	12.2%

**Table 4:** Frequency in number and % of patients with positive tests for the presence of herpesvirus.

	Patients	
	Number	%
**total**	**18**	**43.9%**
HSV 1	3	7.3%
CMV	1	2.4%
EBV	17	41.5%

**Table 5:** Frequencies in number and % of patients with one or more than one herpesvirus.

Number of Herpes Viruses-HV	Patients	
	Number	%
3	0	0%
2	3	7.3%
1	15	36.6%
0	23	56.1%

**Table 6:** Number and % of infected patients for each HPV (LR) and herpesvirus, with 95% confidence intervals.

	Patients		95% CI
	Number	%	
**HPV LR**	**36**	**87.8%**	
6/11	33	80.5%	66.0, 89.8%
42	25	61.0%	45.7, 74.3%
43	19	46.3%	32.1, 61.3%
44	5	12.2%	5.3, 25.5%
**HV**	**18**	**43.9%**	
HSV 1	3	7.3%	2.5, 19.4%
CMV	1	2.4%	0.4, 12.6%
EBV	17	41.5%	27.8, 56.6%

**Table 7:** Coinfection with Epstein–Barr Virus (EBV) and Human Papilloma Virus (HPV).

	HPV 6/11	HPV 42	HPV 43	HPV 44
EBV and HPV	36.6%	29.3%	24.4%	4.9%
EBV only	4.9%	12.2%	17.1%	36.6%
HPV only	43.9%	31.7%	22.0%	7.3%
neither	14.6%	26.8%	36.6%	51.2%
χ^2^	11.3	2.72	0.06	15.4
Adjusted *p* (Bonferroni)	<0.004	0.396	1.000	0.038

## Data Availability

The original contributions presented in this study are included in the article/Supplementary Material. Further inquiries can be directed to the corresponding author(s).

## Supplementary Materials

The following supporting information can be downloaded at: https://www.mdpi.com/article/10.3390/biomedicines13102339/s1, Supplementary File with three Figures Supplementary S1–S3. Figure S1: PCR reactions for detection of EBV, after second round of nested PCR. Specific fragments with a size 209 bp. Figure S2: Nonspecific PCR products with size ~590 bp, after nested PCR for detection of BKPyV. Figure S3: Electropherogram after sequencing of the nonspecific PCR products with size ~590 bp, after PCR for detection of BKPyV.

## Abbreviations

The following abbreviations are used in this manuscript:

BKPyV	BK polyomavirus
DNA	Deoxyribonucleic acid
EBV	Epstein–Barr virus
HCMV	Human cytomegalovirus
HPV	Human papillomaviruses
HNCs	Head and neck cancers
HSVs	Herpes Simplex Viruses
HSV1	Herpes Simplex Virus 1
HSV2	Herpes Simplex Virus 2
PCR	Polymerase chain reaction
SCC	Squamous Cell Carcinoma
