# Supplementary material for: Detection of Viruses with Oncogenic and Oncomodulatory Potential in Head and Neck Tumors—External Auricle
Source: Biomedicines. 2025 Sep 25;13(10):2339. doi: 10.3390/biomedicines13102339 (PMC12561493; doi:10.3390/biomedicines13102339)
Supplement: Supplementary file 1 [file biomedicines-13-02339-s001.zip › biomedicines-3816980-supplementary.pdf]

Supplementary file:

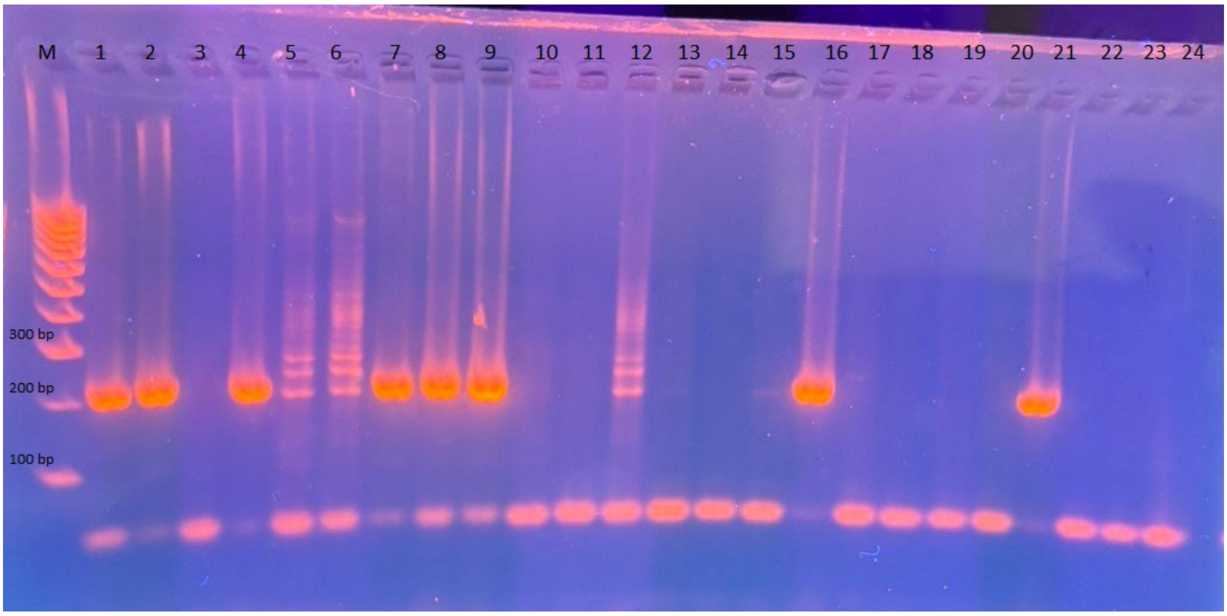

Supplementary Figure S1. PCR reactions for detection of EBV, after second round of nested PCR. Specific fragments with a size 209 bp.

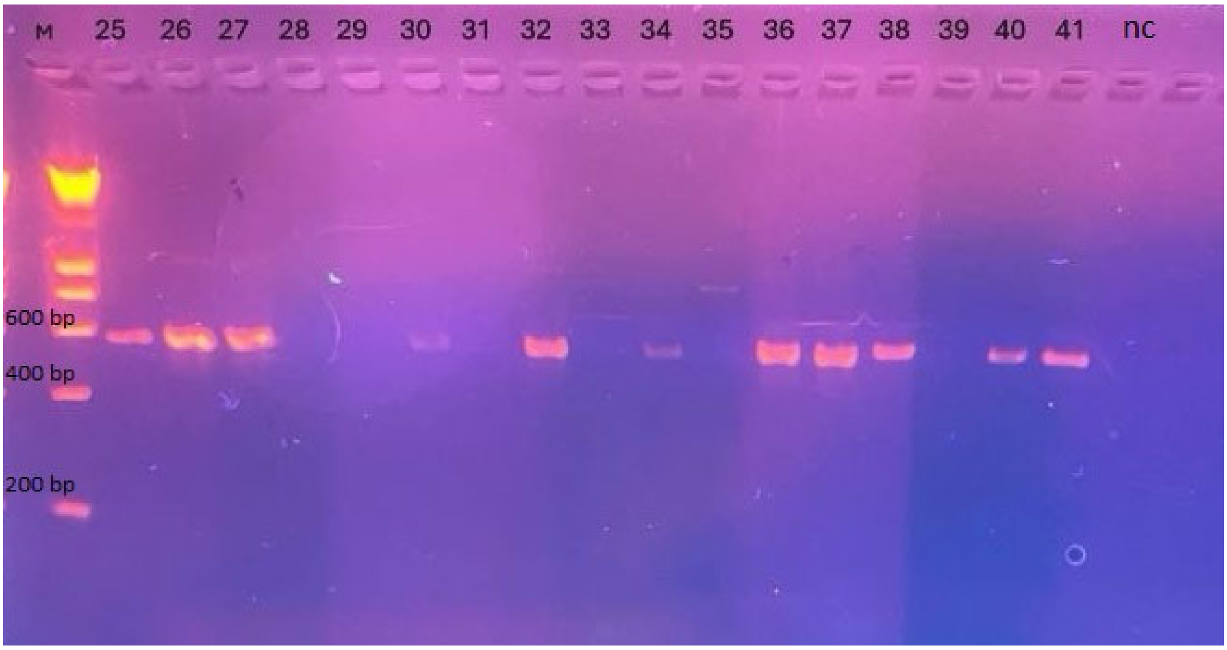

Supplementary Figure S2. Nonspecific PCR products with size ~590 bp, after nested PCR for detection of BKPyV.

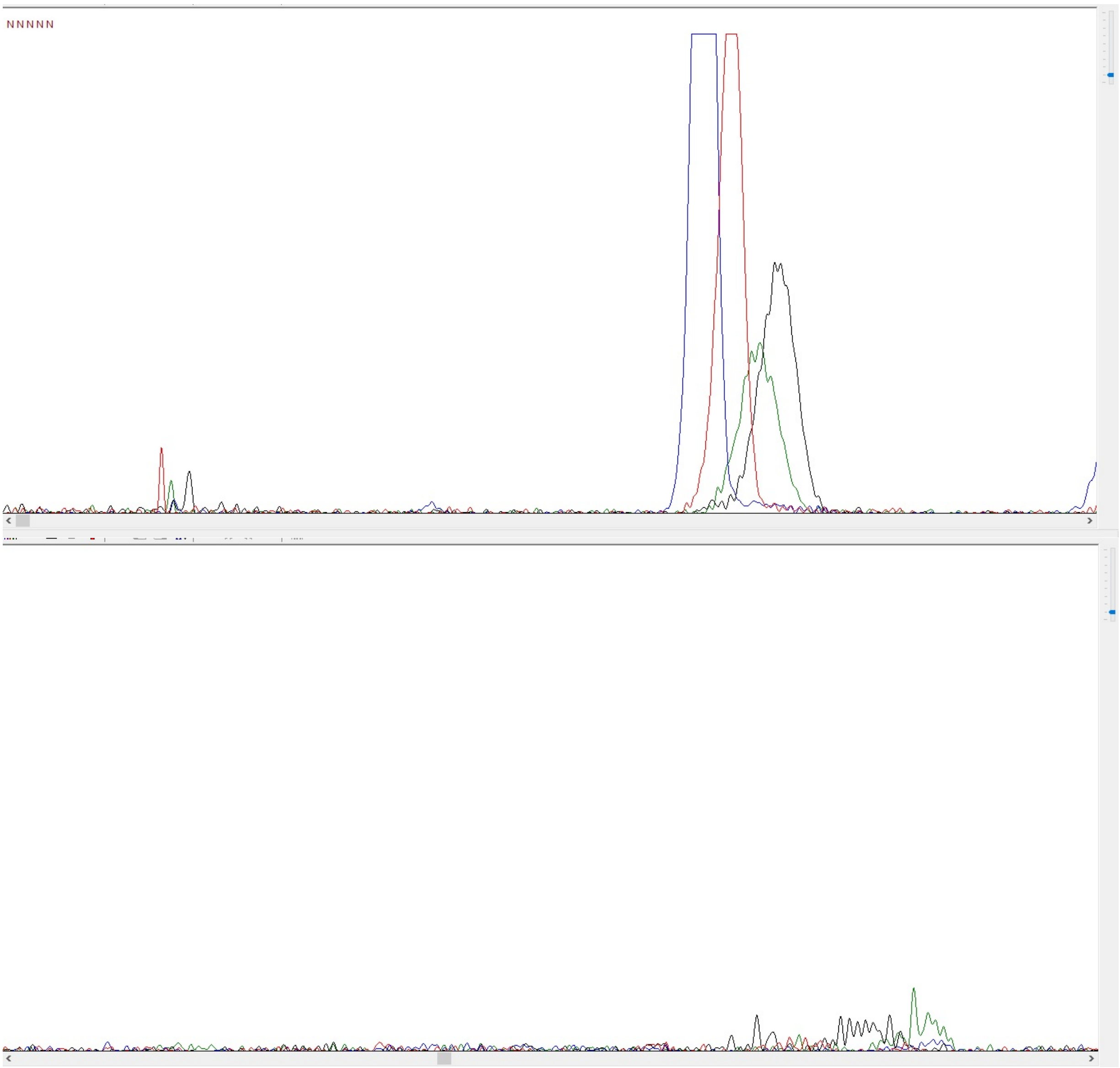

Supplementary Figure S3. Electropherogram after sequencing of the nonspecific PCR products with size ~590 bp, after PCR for detection of BKPyV.
